# Expression of Sex Hormones in Oral Squamous Cell Carcinoma: A Systematic Review on Immunohistochemical Studies

**DOI:** 10.7759/cureus.25384

**Published:** 2022-05-27

**Authors:** Saranya R, Chandini R, Khadijah Mohideen, Pooja N Adtani, Vijayanirmala Subramani, Murali Balasubramaniam

**Affiliations:** 1 Oral and Maxillofacial Pathology, Sathyabama Dental College and Hospital, Sathyabama Institute of Science and Technology, Chennai, IND; 2 Basic Medical and Dental Sciences, College of Dentistry, Gulf Medical University, Ajman, ARE; 3 Oral and Maxillofacial Pathology, Faculty of Dental Sciences, Sri Ramachandra Institute of Higher Education and Research, Chennai, IND

**Keywords:** oral squamous cell carcinoma, immunohistochemistry, alcohol, smoking, areca nut, tobacco, breast cancer, estrogen receptors, hpv, sex hormones

## Abstract

Oral squamous cell carcinoma (OSCC) is the most widespread oral malignancy. In the western world, smoking and alcohol remain the most common risk factors, whereas smokeless tobacco and areca nut consumption are the prevalent etiological factors in South Asia. Numerous markers were studied till date to identify the disease. However, the role of sex hormones in OSCC is poorly defined. Hormonal receptors play a role in breast cancer, prostate cancer, and human papillomavirus (HPV) driven oropharyngeal cancers. It is proven that the expression of estrogen receptor-α improved the overall survival of the patient with HPV-positive oropharyngeal cancer. Thus, this review article aims to determine the expression of sex hormones by immunohistochemistry in OSCC.

This systematic review was performed with Preferred Reporting Items for Systematic Reviews and Meta-Analyses (PRISMA) Statement Criteria 2020. Relevant articles were collected from the electronic database in PubMed and Cochrane till 2021. Immunohistochemical studies on sex hormones and their role in OSCC published in English literature were included. We excluded case reports, case series, and abstract-only articles. The sample size of the selected studies varied, and these studies measured the parameters such as sex hormones and OSCC. Furthermore, all the studies used different sex hormones and their association with oral cancer through the immunohistochemical process. Thus, this review summarizes that sex hormones influence the biopathology of oral cancer and have a prognostic significance in the tumor subgroups.

## Introduction and background

Oral squamous cell carcinoma (OSCC) is the most commonly occurring cancer in the head and neck region, with an estimate of 6,50,000 new cases worldwide every year [1]. The mortality rate of oral cancer is high, with 4,50,000 deaths per year. Several etiological factors, such as smoking and alcohol consumption, play a role in causing the disease with the occurrence being high in males [2]. Numerous markers, such as cytokeratin 19 (CK19), cytokeratin 8 (CK8), beta 2-microglobulin, cluster of differentiation (CD) 44, CD80, p53, C-X-C chemokine receptor type 4 (CXCR4), CC chemokine receptor 7, p16, pRb, human papillomavirus deoxyribonucleic acid (HPV DNA), and E6/7 viral genes, play an important role in predicting the metastasis and prognosis in head and neck squamous cell carcinoma (HNSCC) patients [3]. Sex hormones were more frequently detected to play a major role in breast cancer, prostate cancer, and oropharyngeal tumors, but their involvement in HNSCC remains controversial [4]. Multiple studies were undertaken to define the expression of hormonal receptors in oral cancer and its potential clinical significance. Cancers with different pathological characteristics and treatment responses depend on the expression of sex hormone receptors [5]. Currently, several hormonal therapies are available to treat specific tumors such as androgen-dependent prostate cancer (e.g., enzalutamide) and human epidermal growth factor receptor 2 (HER2) positive breast cancers (e.g., tamoxifen). Howell et al. used immunohistochemistry (IHC) to detect androgen receptors (ARs) in the normal oral mucosa [6].

Estrogen receptor-α (ER-α) and estrogen receptor--β (ER-β) are located in either nucleus or cell cytoplasm of the normal mucosal cells and have a non-genomic effect. AR accumulation in the cytoplasm is associated with an increased risk of metastasis [7]. Aromatase, known as estrogen synthase, is responsible to convert androgen to estrogen. The expression of aromatase is correlated with increased ER-α/ER-β expression, reduced progesterone receptor (PR) expression, and increased cell proliferation and transcription of HPV oncogenes [8]. Interestingly, the expression of ER-α improved the overall survival of the patient with HPV-positive oropharyngeal cancer [9].

This systematic review aimed to determine the expression of sex hormones in OSCC through immunohistochemical studies and to conclude its protagonist action in the prognosis of the disease.

## Review

Methodology

The literature search was carried out on IHC studies that evaluated the role of sex hormones as markers in OSCC from 1984 to 2021 using PubMed and Cochrane databases.

Search strategy

Keywords and Search Terms

The search for a qualitative method of research was conducted using the SPIDER framework (Sample, Phenomenon of Interest, Design, Evaluation, and Research type). The keywords were discovered, and a list of synonyms and MeSH terms was developed to search. A Boolean operator search was established, and MeSH terms were as follows (Sex hormones AND OSCC), (IHC AND Sex hormones), (ER AND OSCC), (IHC AND AR).

Inclusion and Exclusion Criteria

The articles that were published in English from the year 1984 to 2021 were included in PubMed and Cochrane databases. A total of 516 articles were identified. This review included original research, observational studies, and randomized control study in human samples, which were based on the expression of various sex hormones by the IHC method. Studies were excluded if (i) they were in other languages, or were case reports, abstracts only articles, duplicates, and letters to the editor, (ii) were irrelevant, and (iii) used molecular techniques such as polymerase chain reaction (PCR), enzyme-linked immunosorbent assay (ELISA), and western blot. The articles were thoroughly analyzed based on the eligibility criteria, and later data were extracted.

Review Process

**Figure 1:**
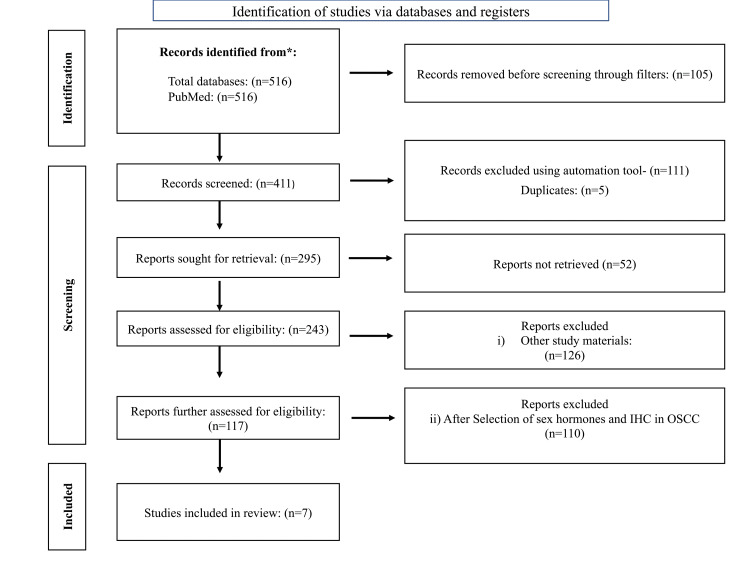
PRISMA protocol: systematic review of included articles PRISMA, Preferred Reporting Items for Systematic Reviews and Meta-Analyses

All the reviewers were involved in the entire review process. This included the various stages of the review process such as screening, data extraction, and observations in the study. The articles obtained by the Boolean search were screened for abstracts, and titles that encountered the inclusion criteria were organized. Furthermore, Preferred Reporting Items for Systematic Reviews and Meta-Analyses (PRISMA) 2020 was used in the process (Figure 1).

Results

After the initial electronic search, 516 articles were identified for the review process, of which 105 were marked as ineligible by using filters. Following this, 116 articles were removed using an automation tool for duplicate removal. Finally, after reviewing the abstracts and the full texts, seven articles were selected and included in the process.

Quality Assessment and Risk of Bias

The quality of the selected studies was assessed using The Newcastle-Ottawa Scale (NOS) for assessing the quality of nonrandomized studies, which is performed based on the following three domains

1. Selection

2. Comparability

3. Outcome

The study is awarded a maximum of one star for each domain. In addition, if the studies get a total score of less than 6, then the domain was deemed as “potential risk bias." On the other hand, if the score is more than 6, the study is deemed to have a “low risk bias.” The full text of seven articles that met the criteria was assessed. Out of seven articles, two articles had a high risk of bias and the rest had a low risk (Table 1).

**Table 1:** Assessment of quality and the risk of bias (NOS scale) *Presence of criteria. ^a^A score of less than 6 indicted potential risk bias and a score of more than 6 indicated a low risk bias. NOS, Newcastle-Ottawa Scale

Description	Selection				Comparability		Outcome			Score^a^
Study	Representativeness of the exposed cohort	Selection of controls	Exposure	Outcome interest not present at the start	Comparability of cohorts based on the design	Comparability of cohorts based on the analysis	Assessment of outcome	Was follow-up long enough for outcomes to occur	Adequacy of follow-up of cohorts	
Doll et al. [10]	*	-	*	-	*	*	*	-	-	5
Chang et al. [11]	*	*	*	-	*	-	-	*	*	6
Colella et al. [12]	*	-	*	-	*	*	*	*	-	6
Koenigs et al. [13]	*	*	-	*	*	-		*	-	5
Lukits et al. [14]	*	*	*	*	-	-	*	*	*	7
Marocchio et al. [15]	*	-	*	*	*	*	-	*	*	7
Mohamed et al. [16]	*	*	*	*	*	-	*	*	*	8

Data Extraction

Finally, the eligible articles were categorized, and the data were extracted. The following items were extracted from each included study: name of the title, authors, journal details, participants, age, sample size, markers used, study method, and area of expression. All the authors screened the available records. As a result, the following data were collected: participants, age, no. of patients, groups, markers used, study method, immunoreactive cases, and area of expression. The study results were tabulated and are given in Table 2.

**Table 2:** Summary of study articles AR, androgen receptor; ER-α, estrogen receptor alpha; ER-β, estrogen receptor beta; HPV, human papillomavirus; IHC, immunohistochemistry; FAK, focal adhesion kinase; OSCC, oral squamous cell carcinoma; PR, progesterone receptor; PCR: polymerase chain reaction

Title	Authors	Journal Details	Participants	Age	No. of Patients	Groups	Markers Used	Study Method	Immunoreactive Cases	Area of Expression	Study Result
Prognostic Significance of ER-α in OSCC	Doll et al. [10]	Cancers (Basel), Volume 13, Issue 22, p. 5763	Primary tumor and primary lymph node metastasis	27-96 yrs	316	Male: 111; female: 205	ER-α	IHC	primary tumor: 11/302; primary lymph node metastasis: 5/52	Nuclear staining	Decreased ER-α expression in male patients and evaluates the aggressiveness
Regulation of ER-α function in OSCC cells by FAK signaling	Chang et al. [11]	Endocrine-Related Cancer, Volume 21, Issue 4, pp. 555-65	Benign and malignant tumors	-	31	-	ER-α	IHC and cell culture	Benign: 0/11; malignant tumors: 12/21	Nuclear staining	FAK regulates ER-α function in OSCC cells
Expression of sexual hormones receptors in OSCC	Colella et al. [12]	International Journal of Immunopathology and Pharmacology, Volume 24, Issue 2, pp. 129-32	OSCC	38-74 yrs	20	Male: 14; female: 6	ER-α and AR	Reverse transcription PCR	10/20	Nuclear staining	The length of the AR modifies the AR transactivation activity in different cell types
Association of ER-α expression with survival in oropharyngeal cancer following chemoradiation therapy	Koenigs et al. [13]	Journal of the National Cancer Institute, Volume 111, Issue 9, pp. 933-42	HPV-positive oropharyngeal cancer and primary chemoradiation therapy	33-66 yrs	515	Male: 174; female: 41	ER-α	IHC	HPV-positive oropharyngeal cancer: 103/177; primary chemoradiation therapy: 111/515	Nuclear staining	HPV-positive oropharyngeal squamous cell carcinoma has an excellent prognosis with overall survival rates greater than 80%
Molecular identification, expression, and prognostic role of estrogen and PR in head and neck cancer	Lukits et al. [14]	International Journal of Oncology, Volume 30, Issue 1, pp. 155-60	OSCC	42-86 yrs	69	Male: 56; female: 13	ER-α, β and PR	IHC and PCR	Coexpression of estrogen and PR: 27/69	Nuclear staining	Coexpression of estrogen and PR = progression of the lesion
Estrogens and AR in OSCC	Marocchio et al. [15]	Acta odontologica Scandinavica, Volume 71, Issue 6, pp. 1513-9	OSCC	45-65 yrs	60	Male: 30; female: 30	AR Aromatase ER- α ER- β	IHC and cell culture	AR:16/60; aromatase: 8/60; Er-α: 5/60; ER-β: 23/60	Nuclear staining	Aromatase expression: poorly differentiated
Expression of hormone receptors in oropharyngeal squamous cell carcinoma	Mohamed et al. [16]	European Archives of Oto-Rhino-Laryngology, Volume 275, Issue 5, pp. 1289-300	Oropharyngeal cancer and HPV-positive cases	-	199	Male: 147; female: 52	AR ER PR	IHC	AR: 31/199; ER: 126/199; PR: 54/199	Nuclear staining	AR expression: invasive front of the tumor in HPV-related tumors, whereas PR expression is more in HPV-negative tumors

Characteristics of the Study

These studies were reported under the guidelines of the Strengthening the Reporting of Observational Studies in Epidemiology (STROBE) Statement. All the studies included from 1984 to 2021 were considered, among which seven articles met the criteria, of which all were longitudinal studies with OSCC as a study sample. All the included cohort studies had participants with the primary tumor, primary lymph node metastasis, benign and malignant tumors, HPV-positive oropharyngeal cancer, and primary chemoradiation therapy. The included studies had both male and female cohorts, wherein different-sex hormones were used in the sample. The sex hormone markers showed immunopositivity in the nuclear area.

Results of Individual Studies Based on the Outcome

Doll et al. concluded that patients with positive ER-α expression had significantly lower overall and relapse-free survival (RFS) than those with negative ER-α expression. For the primary tumor and primary tumor/primary lymph node metastasis cohorts with ER-positive primary tumors, ER expression was significantly associated with overall survival (OS) and relapse-free survival in males but not in females [10]. There was a significant correlation between positivity and tumor localization. ER positivity in the primary tumor was correlated to the International Union against Cancer (UICC) TNM staging. The majority of cases were in stage IV and had a higher rate of bone infiltration [17].

Marocchio et al. demonstrated that ER-β was expressed in almost 40% of the cases and AR in 26%. However, AR expression presented statistically significant differences (p=0.023) between genders, four (13.3%) cases in women and 12 (40%) cases in men [15].

Lukits et al. reported a relatively high incidence of functional receptor expression (co-expression of ER and PR) in head and neck cancer regardless of the location of the tumor anatomy. The expressive number of cases expressing ER and PR indicate tshat hormones do not influence a patient's survival but influence the progression of the lesion [14].

Koenigs et al. concluded that ER positivity in HPV-positive oropharyngeal cancers was associated with improved mortality on a general, disease-specific, progression-free, and relapse-free basis; statistically significant associations were maintained when clinical risks were adjusted, including the HPV status of the individual [13].

Discussion

Oral cancer is a leading problem worldwide, increasing OSCC incidence by around 2-6% every year [13]. It is a multifactorial and multistep process. The endocrine milieu is a vital factor in tumor progression, especially in tissues expressing the receptors such as the breast and prostate. Oral cancer is twice as common in men as in women. This difference may be related to the use of alcohol and tobacco, which is a major risk factor seen more commonly in men than in women. Although there is a significant influence of western culture upon the lifestyle, the incidence rate is less in females as women are spared due to the defense mechanism associated with the hormones and specific metabolic activity [14].

Egloff et al. reported a crosstalk between ERs and epidermal growth factors, which promotes tumor progression and poor prognosis [18]. Chan and Reade while analyzing the role of sex hormones in oral cancer observed that the hormones in an oral cancer patient are metabolized differently as compared to a healthy individual [19]. Other hormones such as luteinizing hormone, follicle-stimulating hormone, and prolactin also play a role in oral cancer and prove an alteration in the pituitary-adrenal-testicular axis. Similarly, Bauernhofer et al. reported that the prolactin receptor is an independent factor and affects the overall survival of patients diagnosed with head and neck cancer [20].

This systematic review aimed to analyze the role of sex hormones and their expression through IHC in OSCC. Therefore, this review summarized the key elements based on the published literature. Many research teams focused on the role of hormone receptors in hormone-dependent tissues. Lukits et al. were the first to report the role of ERs (both isoforms α and β) and progesterone receptors in head and neck cancer, laryngeal cancer, and oral cancers. More than 50% of cases showed positivity for messenger ribonucleic acid (mRNA) and protein expression of receptors [14].

Three primary mechanisms involved in tumorigenesis are receptor-mediated hormonal activity leading to cellular proliferation, increased mutation rates through cytochrome P450-mediated metabolic activation causing genotoxic effects, and induction of aneuploidy. Similarly, androgen and its receptors influence the transcription and translation process in normal cell development and differentiation [21]. Experimental studies show that androgen modulates the proto-oncogene expression (C-Myc) and apoptotic pathway (Bcl-2) [22,23]. Suba et al. [24] hypothesized that a deficiency of estrogen in postmenopausal women causes OSCC.

The clinical significance of sex hormones is addressed in many studies. Interestingly, the upregulation of ARs reduced the overall survival of patients with oral cancer, which was driven through micro RNA-21 [25]. However, there was a reduced disease-specific survival (p=0.001) associated with progesterone expression [16]. ER-α is associated with improved overall survival of HPV-positive oropharyngeal cancer and is probably influenced by the apolipoprotein B mRNA editing enzyme catalytic polypeptide (APOBEC) mutational signature, making these tumors more immunogenic [26]. In vivo, anti-estrogen therapies in transgenic mice against cervical cancer showed positive results. The expression of both ER-β and submaxillary gland androgen-regulated protein 3 (SMR3A) had a poor prognosis. This observation shows that ER-α activates multiple pathways in oral cancer to play a protective role or promote resistance [27].

## Conclusions

This review highlights that sex hormones influence the biopathology of oral cancer and have a prognostic significance in tumor subgroups. Hormonal receptors were detected to play a major role in breast cancer, prostate cancer, and oropharyngeal tumors, but their involvement in HNSCC remained controversial. The expression of ER, AR, and PR need to be evaluated with prudence because of the probability of variation in clinicopathological features and their association with different expression patterns in OSCC. Estrogen is known to be carcinogenic, and multiple mechanisms are involved in tumor promotion. Furthermore, estrogen has been shown to cause chromosomal instability, which leads to aneuploidy and the development of oral cancer. Lastly, ER expression could be regarded as a seldom risk factor for OSCC, and PR expression seems to be not relevant for the development of OSCC. Gene profiling techniques combined with molecular techniques could allow the interpretation of comprehensive studies in the future to diagnose and prevent the progression of OSCC.
